# Ursodeoxycholic Acid Is Conjugated with Taurine to Promote Secretin-Stimulated Biliary Hydrocholeresis in the Normal Rat

**DOI:** 10.1371/journal.pone.0028717

**Published:** 2011-12-14

**Authors:** Miriam Úriz, Elena Sáez, Jesús Prieto, Juan F. Medina, Jesús M. Banales

**Affiliations:** Division of Gene Therapy and Hepatology, CIMA Clinic and School of Medicine, University of Navarra, Centro de Investigación Biomédica en Red en el Área temática de Enfermedades Hepáticas y Digestivas, Pamplona, Spain; Université Paris Sud, France

## Abstract

**Background & Aims:**

Secretin induces bicarbonate-rich hydrocholeresis in healthy individuals, but not in untreated patients with primary biliary cirrhosis (PBC). Ursodeoxycholic acid (UDCA) – the first choice treatment for PBC – restores the secretin response. Compared with humans, secretin has poor effect in experimental normal-rat models with biliary drainage, although it may elicit hydrocholeresis when the bile-acid pool is maintained. In view of the benefits of UDCA in PBC, we used normal-rat models to unravel the acute contribution of UDCA (and/or taurine-conjugated TUDCA) for eliciting the biliary secretin response.

**Methods:**

Intravascular and/or intrabiliary administration of agonists and inhibitors was performed in normal rats with biliary monitoring. Secretin/bile-acid interplay was analyzed in 3D cultured rat cholangiocytes that formed expansive cystic structures with intralumenal hydroionic secretion.

**Results:**

*In vivo*, secretin stimulates hydrocholeresis upon UDCA/TUDCA infusion, but does not modify the intrinsic hypercholeretic effect of dehydrocholic acid (DHCA). The former effect is dependent on microtubule polymerization, and involves PKCα, PI3K and MEK pathways, as shown by colchicine (i.p.) and retrograde biliary inhibitors. *In vitro*, while secretin alone accelerates the spontaneous expansion of 3D-cystic structures, this effect is enhanced in the presence of TUDCA, but not UDCA or DHCA. Experiments with inhibitors and Ca^2+^-chelator confirmed that the synergistic effect of secretin plus TUDCA involves microtubules, intracellular Ca^2+^, PKCα, PI3K, PKA and MEK pathways. Gene silencing also demonstrated the involvement of the bicarbonate extruder Ae2.

**Conclusions:**

UDCA is conjugated in order to promote secretin-stimulated hydrocholeresis in rats through Ae2, microtubules, intracellular Ca^2+^, PKCα, PI3K, PKA, and MEK.

## Introduction

Primary bile generated at the canaliculi is fluidized and alkalinized along the intrahepatic biliary tract, a process that is normally stimulated by secretin [1], [2]. In humans, secretin-stimulated bile flow may account for up to 40% of the total bile output from the liver. Previous *in vivo* experiments in an animal model of infused normal rat with biliary drainage, together with data obtained in cultured normal-rat cholangiocytes, indicated that the Cl^−^/HCO_3_^−^ anion exchanger 2 (Ae2) mediates biliary secretion of bicarbonate in response to secretin [3]. Bicarbonate concentration then serves as an osmotic driving force for aquaporin 1 to mediate the efflux of water, leading to increased hydrocholeresis [4]. For secretin to exert these effects in normal live rats we found that the bile-acid pool needs to be maintained by, for instance, continuous infusion of taurocholic acid (TCA) [3]. Ursodeoxycholic acid (UDCA) is a more hydrophilic dihydroxy bile acid that is considered to possess superior pharmacological properties, being currently used as the first choice treatment for a number of cholestatic liver diseases, particularly primary biliary cirrhosis (PBC) [5]–[7]. Among numerous positive effects of UDCA like its ability to increase the hydrophilicity in the pool of bile acids, and its immunomodulatory and cytoprotective properties, UDCA is known to stimulate bicarbonate-rich hypercholeresis in humans [6], [8]. The benefit of a hydrocholeresis that is particularly rich in bicarbonate has recently been enlightened by the attractive “biliary bicarbonate umbrella” hypothesis [9], [10]. Earlier positron-emission tomography (PET) studies using labeled bicarbonate showed that untreated PBC patients have impaired biliary bicarbonate secretion in response to secretin, a defect that is restored in PBC patients treated for a few months with UDCA [11]. On the other hand, untreated PBC patients exhibit diminished expression of AE2 in the liver, and treatment with UDCA appears to be associated with improved AE2 expression [6], [12], [13].

Experiments carried out in normal-rat models with biliary drainage indicate that UDCA, but not its taurine-conjugate tauroursodeoxycholic acid (TUDCA), may stimulate the production and canalicular secretion of S-nitrosoglutathione (GSNO), which leads to hydrocholeresis in the biliary epithelium [14]. The observation that infusion of TUDCA does not trigger the production of GSNO but still promotes bicarbonate-rich hypercholeresis prompted us to analyze the possible direct effects of TUDCA *versus* UDCA on the biliary epithelium and the relationship of those effects with secretin. Also we compared these effects with the effect of dehydrocholic acid (DHCA) – known to strongly promote canalicular bile flow and microtubule-independent hypercholeresis [15] – in relation with secretin. Our findings both in normal rats and in 3D-cultured cholangiocyte cystic structures unraveled the crucial role of conjugation of UDCA for its concerted action with secretin to promote hydrocholeresis. The mechanisms involved in this concerted action include microtubules and Ae2, as well as intracellular Ca^2+^, protein kinase Cα (PKCα), mitogen-activated protein kinases/extracellular-signal regulated kinases (MAPK-ERK1/2) kinase (MEK or MAP2K), phosphoinositide 3-kinase (PI3K), and protein kinase A (PKA) signaling pathways.

## Materials and Methods

### Direct biliary monitoring in infused normal rat

Bile flow was monitored in our already described animal model [3], in which normal live rats are intravenously infused with secretin and/or bile acids, and the bile duct may receive different inhibitors. Briefly, midline abdomen incision in anesthetized normal male Wistar rats (∼250 g) was followed by cannulation of the common bile duct. Then the iliac vein was cannulated for continuous infusion (2 mL/h) of 0.9% NaCl solution with 20 mM of either UDCA, TUDCA (both from Sigma-Aldrich), or DHCA (Calbiochem). Fifteen minutes after starting bile-acid infusion, saline solutions (0.2 mL) with either the specific inhibitor of Ca^2+^-dependent (conventional) PKCα Gö6976 (Calbiochem; 1 µM), the MEK inhibitor U0126 (Promega; 10 µM), or the PI3K inhibitor wortmannin (Fluka Biochemika; 100 nM), were administered intrabiliary (through retrograde fluxes) and let to stand in the bile duct for 20 min. Meanwhile, a bolus of rat secretin (RayBiotech; 40 nmol) was infused in saline solution (0.12 mL) via the iliac vein 5 min after retrograde fluxes, and the biliary cannula was removed 15 min afterwards, bile being collected in 1.5-mL tubes for the first 5 min. In some rats microtubule polymerization was blocked by colchicine (Sigma-Aldrich), administered intraperitoneally (2.5 mg/kg b.w in 0.5 mL PBS) 150 min before bile-acid infusions. The experimental protocol was approved by the University of Navarra Animal Care Committee (approval ID: 065-09).

### Cholangiocyte culture

Normal-rat cholangiocytes were obtained by isolating intrahepatic bile-duct units from male Wistar rats and culturing them on a rat-tail collagen monolayer with an enriched DMEM/F-12 medium, as described for mouse cholangiocytes [16]. For 3D-culture, clusters of rat cholangiocytes were grown between two layers of type I rat tail collagen (1.5 mg/mL) plus 10% Matrigel (both from BD Biosciences), and maintained in enriched DMEM/F-12 medium. Under these conditions, clusters of cholangiocytes spontaneously form cystic structures that expand over time as a consequence of hydroionic secretion to the cyst lumen [17]. Cystic structures were assessed in an inverted light microscope for changes in their expansion in response to 1 µM secretin for 30 min, with or without the presence of 200 µM of bile acids (UDCA, TUDCA and DHCA, as well as TCA). Positive effects were further assessed in the presence of different inhibitors: 0.05 µM colchicine, 100 nM wortmannin, 10 µM U0126, 1 µM Gö6976, 30 µM of the calcium chelator BAPTA (Sigma-Aldrich), and 10 µM of the PKA inhibitor H89 (Sigma-Aldrich). Each inhibitor was added 30 min before and during secretin stimulation for additional 30 min. Cyst expansion was assessed as the circumferential area of the cyst before and after the stimuli and/or inhibition, with the Image J software (National Institutes of Health, Bethesda, MD). Data obtained were expressed as a percentage of cystic-area expansion.

### Gene silencing

Small interfering RNA (siRNA) rAe2 (GGUGUGGACGAGUACAACGUU), against rat Ae2 or control siRNA (GGUGUGGACGAGUACAAUGUU), reported to target human but not rat Ae2 [3], were added to the medium (50 nM each) 1 day before monitoring the cystic structures, as previously reported [18].

### Statistics

Values are shown as mean ± SD or SEM where indicated. The nonparametric Mann-Whitney test was used when two groups were compared. For more than two groups, the nonparametric Kruskal-Wallis test followed by Dunns *post-hoc* test or parametric ANOVA with Bonferroni *post-hoc* test were used. Differences were considered statistically significant when two-tailed *P* values were <0.05 (not adjusted).

## Results

### Effect of secretin on the bile flow in normal rats infused with UDCA, TUDCA or DHCA

**Figure 1:**
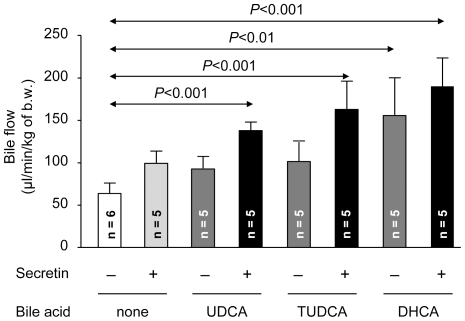
Secretin stimulates bile flow in normal rats infused with UDCA and TUDCA. The choleretic effects of secretin, UDCA, or TUDCA administered alone were not significant, while infusion of DHCA alone stimulated the bile flow of normal rats, with no effect on the secretin response. All relevant differences are indicated (with no statistical difference between secretin-stimulated bile flows in UDCA- and TUDCA-infused animals). Data are shown as mean ± SD.

Secretin administration to normal rats infused with UDCA increased the bile flow compared with baseline bile flow (i.e. in rats without infusion of both secretin and bile acids, Fig. 1). Replacement of unconjugated UDCA with taurine-conjugated TUDCA resulted in similar secretin-stimulated bile flow *versus* baseline values (Fig. 1). Neither UDCA, TUDCA nor secretin alone significantly increased the bile flow (Fig. 1). As previously reported in other animal models [19], infusion of normal rats with DHCA alone induced hydrocholeresis (Fig. 1). However, DHCA-induced choleresis was not affected by secretin (Fig. 1).

### Effects of inhibitors on secretin-stimulated hydrocholeresis in normal rats infused with UDCA or TUDCA

**Figure 2:**
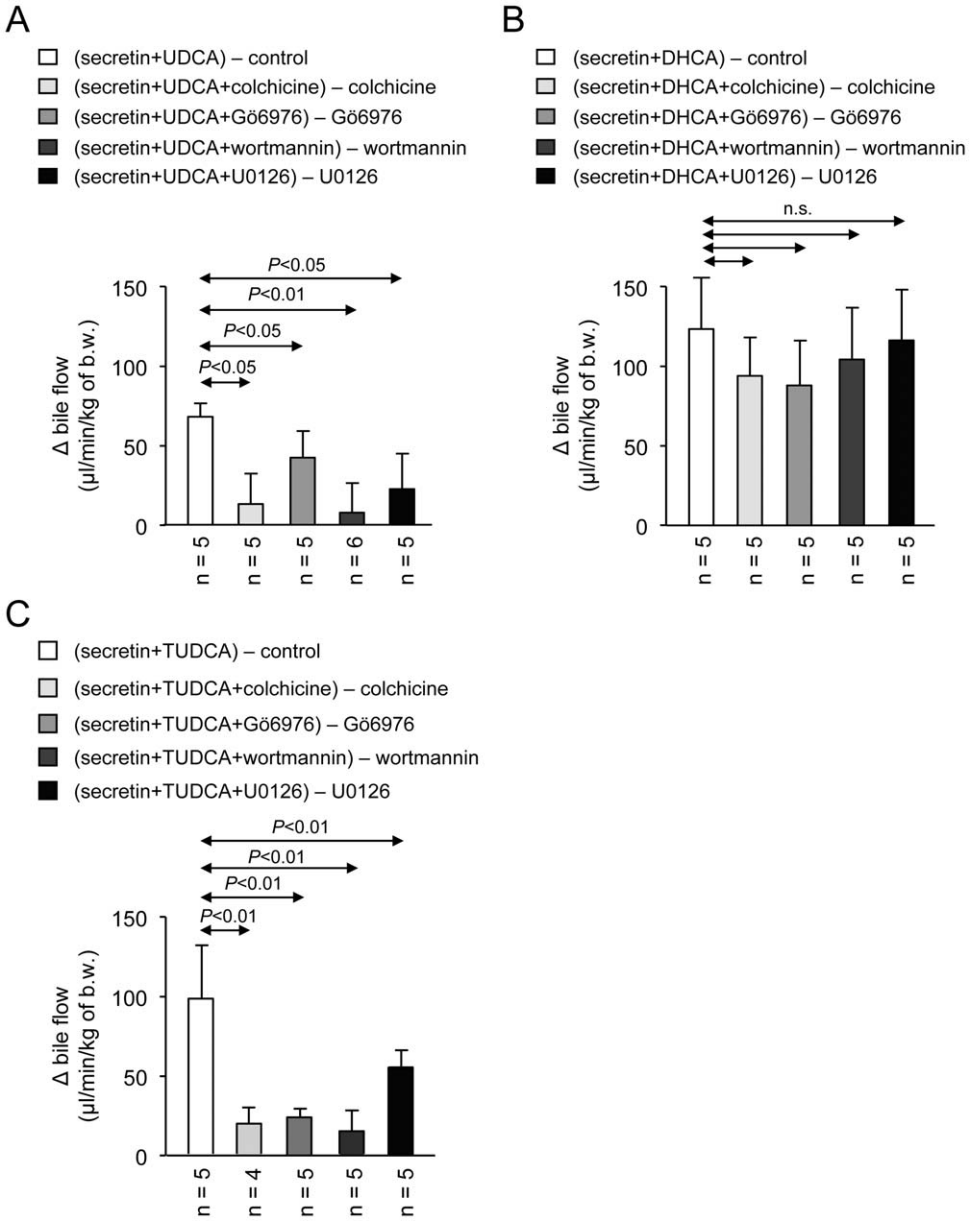
Increased bile flow in rats receiving secretin and either UDCA or TUDCA is dependent on microtubule polymerization, PKC, PI3K and MEK. The increased bile flow in rats receiving secretin plus (A) UDCA or (B) TUDCA was blocked with intraperitoneal administration of colchicine (an inhibitor of microtubule polymerization), intrabiliary administration of the Ca^2+^-dependent PKCα inhibitor Gö6976, intrabiliar wortmannin (a PI3K inhibitor), and intrabiliar U0126 (a MEK inhibitor). (C) On the other hand, the hydrocholeretic effect to DHCA was unaffected by these inhibitors. Increases *versus* respective controls were calculated as indicated. Data are shown as mean ± SD.

Intraperitoneal injection of colchicine (an inhibitor of microtubule polymerization and microtubule-dependent vesicle mobilization) did not modify the basal bile flow (not shown), but did block the secretin-stimulated bile flow in UDCA- and TUDCA-infused animals (Fig. 2A–B). On the other hand, colchicine did not significantly modify the increased bile flow observed in DHCA-infused normal rats, regardless of secretin administration (Fig. 2C).

To elucidate the mechanism by which secretin stimulates the bile flow in the UDCA- and TUDCA-infused normal rats, intrabiliary administration of inhibitors was employed. We therefore tested the role of PKCα (an important Ca^2+^-dependent kinase involved in processes of bile acid-dependent secretion) [20], by using the inhibitor Gö6976. While intrabiliary administration of Gö6976 alone did not affect baseline bile flow (data not shown), it attenuated the stimulatory effect of secretin in the animals infused with either UDCA or TUDCA (Fig. 2A–B). Gö6976, however, did not diminish the bile-flow increases in the DHCA-infused normal rats with and without secretin administration (Fig. 2C).

We analyzed the role of two additional kinases, PI3K and MEK, which are frequently involved in secretory processes in cholangiocytes, through intrabiliary administration of wortmannin and U0126, respectively. While neither wortmannin nor U0126 alone affected the baseline bile flow in normal rats (data not shown), both inhibitors dampened down the increased bile flow in the normal rats receiving secretin plus either UDCA or TUDCA (Fig. 2A–B). On the other hand, none of these inhibitors significantly diminished the bile-flow increases in the DHCA-infused normal rats, regardless of secretin administration (Fig. 2C).

### Effect of secretin on the cystic expansion of 3D-cultured normal-rat cholangiocytes in the presence of bile acids

**Figure 3:**
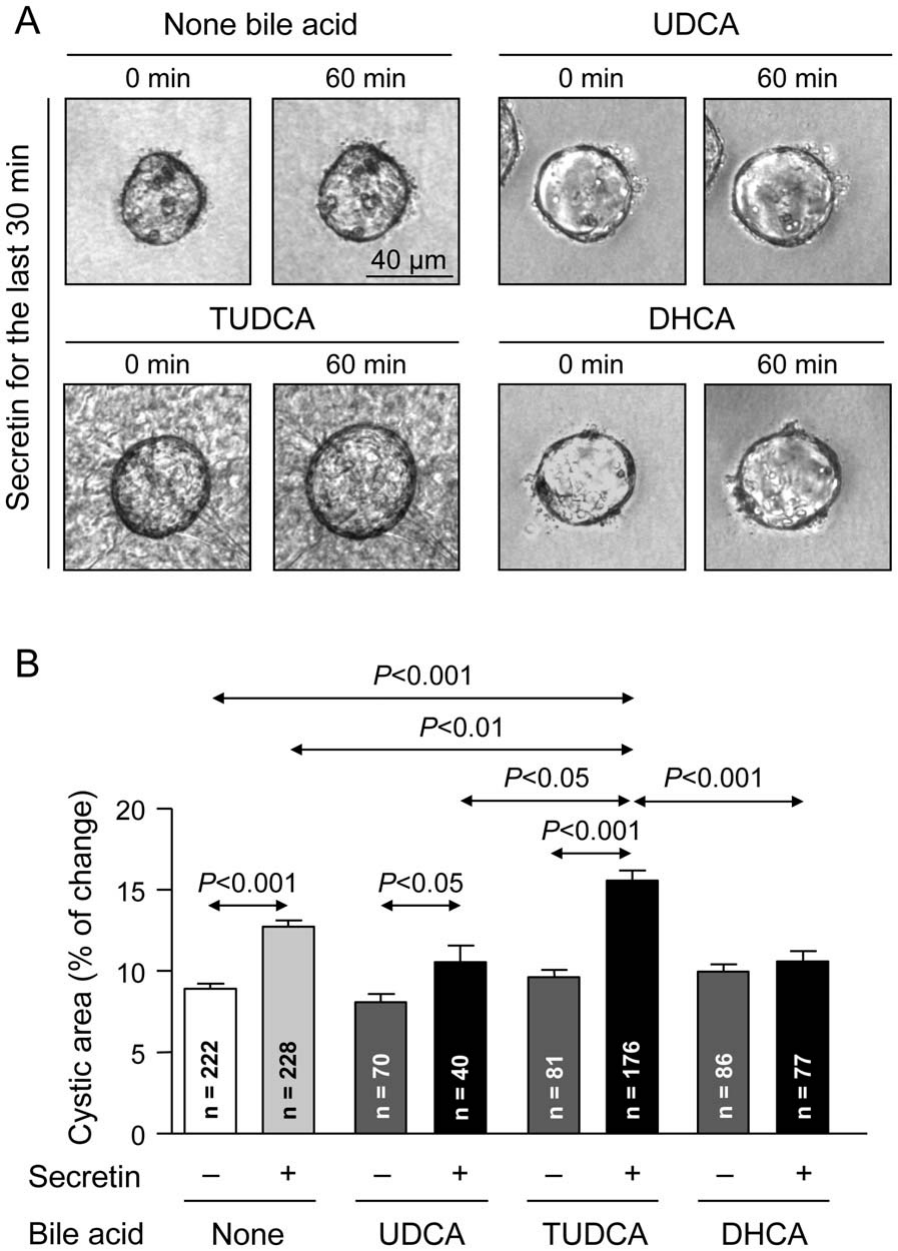
TUDCA promotes secretin-stimulated expansion of 3D-cultured cholangiocyte cystic structures. (A) Representative images of cystic structures incubated for 60 min in the presence or absence of bile acids (UDCA, TUDCA, or DHCA), and with secretin for the last 30 min. (B) While secretin alone stimulated cystic expansion, such an expansion was further accelerated in the presence of TUDCA, but not in the presence of UDCA or DHCA (which rather tended to block the stimulatory effect of secretin). Only comparisons of interest are indicated.

To directly analyze the secretin/bile-acids interplay in cholangiocytes we used the recently described model of cholangiocyte cystic structures in 3D [17]. They expand spontaneously as a consequence of hydroionic secretion to the cyst lumen, and accelerate their expansion rate in the presence of secretin [17]. Indeed, we reproduced these findings and found that administration of secretin alone during 30 min accelerated the expansion of cholangiocyte cystic structures (Fig. 3A–B). The cooperative effect of bile acids on the *in vitro* secretin-stimulated hydrocholeresis was then analyzed by adding either UDCA, TUDCA, or DHCA to the culture medium. At the concentration of 200 µM, TUDCA was found to further accelerate the secretin-stimulated cystic expansion, while the presence of unconjugated UDCA in the culture medium showed no concerted action with secretin (Fig. 3A–B). TUDCA exerted its effect in a dose-dependent manner (Fig. S1). Also, the effect of TUDCA was specific, as TCA (another taurine-conjugated bile acid) did not exhibit any concerted action with secretin (Fig. S2A–B). The presence in the culture medium of 200 µM DHCA (an unconjugated bile acid with intrinsic choleretic effect *in vivo*) did not further accelerate the stimulatory effect of secretin (Fig. 3A–B). And neither TUDCA, UDCA, nor DHCA alone (in the absence of secretin) affected the spontaneous baseline expansion of cholangiocyte cystic structures in our cell culture conditions (Fig. 3A–B). Although higher concentrations of bile acids were also employed, no positive effects were observed on either spontaneous or secretin-stimulated cystic-structure expansion (data not shown).

### Effects of inhibitors on the expansion of 3D-cultured cholangiocyte cystic structures in the presence of TUDCA+secretin

**Figure 4:**
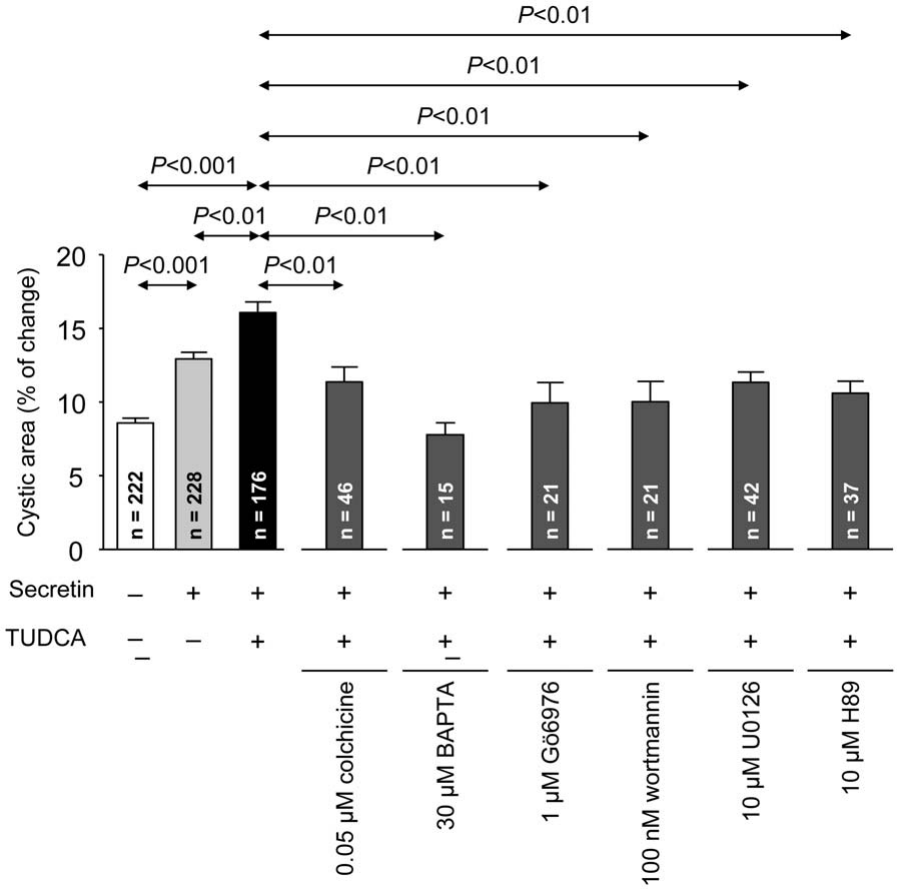
The expansion of 3D-cultured cholangiocyte cystic structures stimulated by the combination of secretin and TUDCA is dependent on microtubule polymerization, intracellular Ca^2+^ and PKCα signaling, and PI3K, PKA and MEK pathways. [TUDCA+secretin]-stimulated cystic expansion was sensitive to colchicine, the Ca_2_^+^-chelator BAPTA, Gö6976, wortmannin, U0126 and the PKA-inhibitor H89. Cystic expansion is expressed as percentage of the area at the end of the experiment (60 min) *versus* initial area (at time 0). Data are shown as mean ± SEM.

In view of the blocking effects observed *in vivo* with colchicine, we assessed the role of microtubule polymerization for the cystic-structure expansion stimulated by secretin+TUDCA. While the presence of colchicine in the culture medium did not modify the baseline expansion (see for the inhibitors alone), it blocked the [secretin+TUDCA]-stimulated expansion of cystic structures (Fig. 4; see Fig. S3B for representative images).

We also tested the role of intracellular Ca^2+^ by adding BAPTA (a chelator of intracellular Ca^2+^) or Gö6976 (the inhibitor of the Ca^2+^-dependent PKCα) to the culture medium. While neither BAPTA nor Gö6976 alone affected the baseline cystic-structure expansion, they both inhibited the cystic expansion stimulated by the combination of secretin and TUDCA (Fig. 4).

Likewise, the involvement of PI3K, MEK, and PKA in cholangiocyte cyst hydrocholeresis was tested by using their respective inhibitors wortmannin, U0126, and H89. These inhibitors each blocked the cystic expansion stimulated by the combination of secretin and TUDCA (Fig. 4). On the other hand, wortmannin, U0126, and H89 alone, i.e. in the absence of secretin and TUDCA, were each found to stimulate the baseline expansion of the cystic structures, suggesting non-specific effects of these inhibitors.

### Effects of Ae2 silencing on the expansion of 3D-cultured cholangiocyte cystic structures

**Figure 5:**
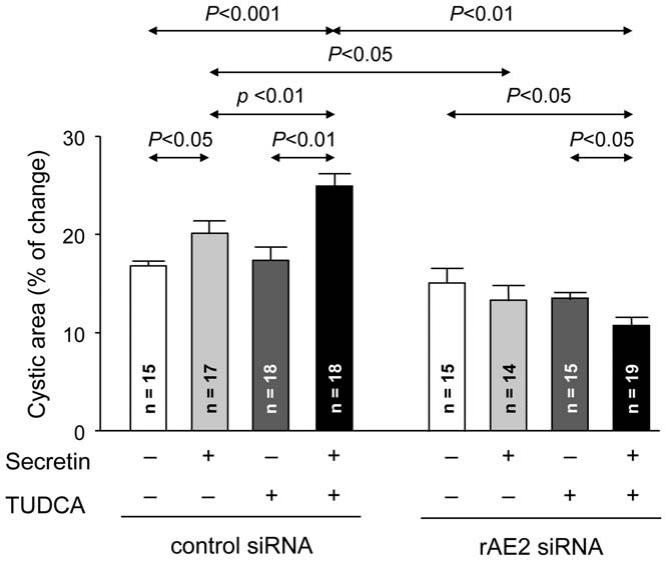
The bicarbonate extruder Ae2 is involved in the concerted hydrocholeretic effect of TUDCA and secretin. While the presence of control siRNA had no effect on the expansion rate of 3D-cultured cholangiocyte cystic structures stimulated by secretin alone or combination of secretin and TUDCA (*left*), the specific rAe2 siRNA against rat Ae2 mRNA blocked all stimulatory effects (*righ*t). Data are shown as mean ± SEM.

Finally, we analyzed (in an additional set of experiments) the role of Ae2 (the Cl^−^/HCO_3_^−^ exchanger that mediates bicarbonate secretion in normal-rat cholangiocytes) [3], for the hydrocholeresis stimulated by secretin+TUDCA in the cystic structures. Control and rAe2 siRNAs were added to the culture medium 24 h before the analysis of cystic hydroionic secretion. As previously reported [17], under these conditions Cy3-labeled siRNAs can directly enter the cholangiocytes (Fig. S4A). Although the presence of control siRNA did not affect the cystic-structure expansion stimulated by secretin and TUDCA, the presence of the siRNA against Ae2 totally blocked cystic expansion under all circumstances (Fig. 5 and Fig. S4B).

## Discussion

**Figure 6:**
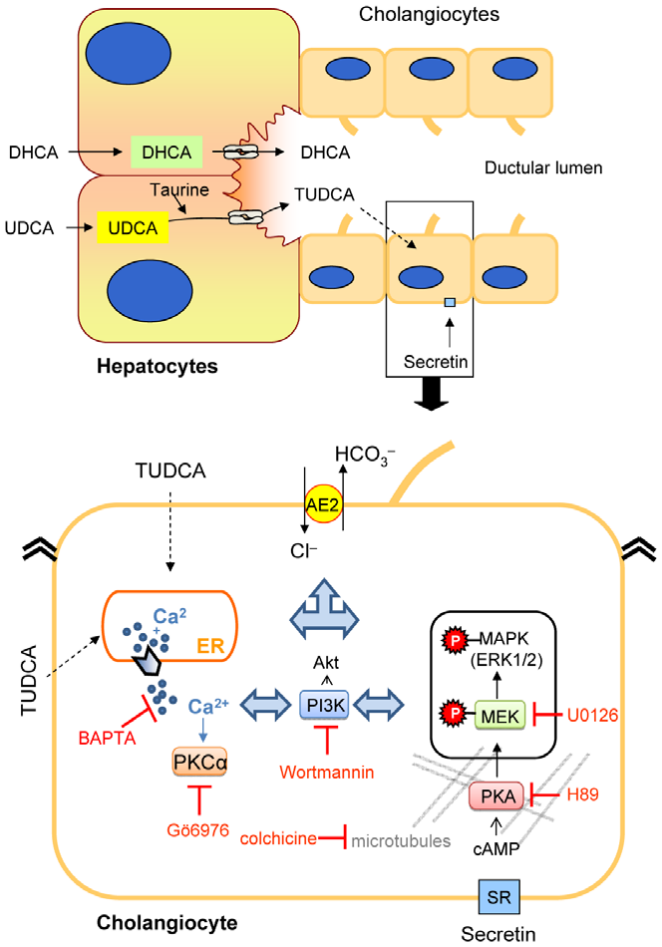
Mechanisms proposed for the TUDCA-mediated secretin-stimulated bile flow in the normal rat. The diagrams summarize the proposed mechanisms for the concerted action of UDCA and secretin that stimulates further the hydrocholeresis in the normal-rat biliary epithelium: UDCA is conjugated with taurine in the hepatocytes and secreted to bile as TUDCA, which may directly access the apical side of cholangiocytes (as well as the basolateral side in the case of an intact animal with enterohepatic circulation). In cholangiocytes, TUDCA may favor the action of secretin through modulation of intracellular Ca_2_^+^ levels, and PKCα and PI3K activation. MEK and cAMP-dependent pathways as well as mobilization of vesicles appear to be also involved. Ae2 has a crucial role for the secretin-stimulated bicarbonate-rich hydrocholeresis by acting as the ultimate extruder of bicarbonate through Cl^−^/HCO_3_^−^ exchange. Neither DHCA nor unconjugate UDCA may exert any of these acute effects on the biliary epithelium, and they promote hydrocholeresis via different mechanisms.

In the hepatobiliary tract, secretin acts in the cholangiocytes (but not in the hepatocytes), stimulating bicarbonate-rich hydrocholeresis [1]. Thus, secretin stimulation constitutes a feasible maneuver to assess the health status of the biliary epithelium. In fact, we previously showed with PET technology that untreated PBC patients have defective biliary bicarbonate secretion in response to secretin administration, a defect that is reversed in patients receiving UDCA for several months [11]. Currently, UDCA is the only approved therapy for PBC [5], [7], [8], and a majority of PBC patients who have initiated the UDCA treatment at early stages of the disease show delayed progression to cirrhosis, fewer complications and even normal life expectancy [5], [7]. Our present data using secretin stimulation in both *in vivo* and *in vitro* normal-rat models unravel the crucial role of conjugation of UDCA for its concerted action with secretin to promote hydroionic secretion at the rat biliary epithelium. The mechanisms involved in this concerted action include microtubules and Ae2, as well as intracellular Ca^2+^, PKCα, PI3K, MEK and PKA signal transduction cascades (summarized in Fig. 6).

Our experimental model of infused normal rat with biliary drainage allows for the analysis of the effects of intravascular and/or intrabiliary administration of molecules over the bile flow [3]. In contrast to the rodent models with bile-ductal hyperplasia (like the bile-duct ligated rat) in which secretin displays manifest biliary effects, the model of normal rat with biliary drainage was shown to require a maintenance of the bile acid pool (for instance through continuous bile-acid infusion) for secretin to achieve significant effects [3]. Here, our model of infused normal rat with biliary drainage was highly convenient to explore *in vivo* the mechanisms involved in the cooperative interplay between secretin and bile acids, particularly UDCA and its taurine-conjugated form TUDCA, at the biliary epithelium. To better dissect such a cooperative interplay observed *in vivo*, we also used our recently described *in vitro* model of 3D-cultured cholangiocyte cystic structures [17], [18]. These cystic structures expand spontaneously over time as a consequence of secretion to the cyst lumen, and are able to accelerate their expansion rate with secretin [17]. Accordingly, they were used to evaluate the concerted action of bile acids with the primary secretin effect, further promoting the hydroionic secretion of functional cholangiocytes.

While *in vivo* infusion of either UDCA or TUDCA allowed for secretin to increase hydrocholeresis (Fig. 1), only TUDCA positively cooperated with the primary effect of secretin *in vitro*, accelerating the expansion rate of cholangiocyte cystic structures *versus* secretin alone (Fig. 3). These data indicate that the biliary hydrocholeretic effect observed with secretin in the UDCA-infused rats involves conjugation of UDCA. In fact, before its canalicular secretion, UDCA is normally conjugated with taurine and glycine (mostly with taurine in the rat) [21], by the enzyme BAAT (bile acid coenzyme A:amino acid N-acyltransferase) located in the peroxisomes [22]. Previous reports indicated that conjugation is relevant for the anticholestatic [23] and cytoprotective [24] effects of UDCA in hepatocytes. Now, we show that conjugation of UDCA is also needed for its cooperation in promoting the hydrocholeretic effect of secretin in cholangiocytes. It should be taken into account that when UDCA is clinically employed for the treatment of cholestatic diseases, it is orally administered in the unconjugated form, but once it reaches the liver through the portal circulation, it is rapidly conjugated in the hepatocyte and secreted to bile, the unconjugated UDCA being virtually undetectable in bile.

In our *in vitro* model of cystic structures, both secretin and bile acids reach cholangiocytes at their basolateral side. In our *in vivo* model of UDCA-infused rats, however, the biliary drainage interrupts the enterohepatic circulation, and biliary-secreted conjugated TUDCA reaches cholangiocytes at their apical side only. In clinical settings like that of PBC patients treated chronically with UDCA, a significant portion of the biliary-secreted conjugated TUDCA may return to blood through the enterohepatic circulation and reach also the basolateral side of cholangiocytes (similarly to our *in vitro* model).

Before UDCA was widely used for the treatment of cholestatic disorders, the semi-synthetic triketo bile acid DHCA was considered a valid therapeutic approach because of its hypercholeretic properties and low toxicity [19]. Our *in vivo* and *in vitro* data indicate that the pronounced hypercholeretic effect of DHCA in the rat is neither related to the cholangiocytes nor modulated by secretin, being seemingly circumscribed to the hepatocytes. The molecular mechanisms by which DHCA exerts its hypercholeretic action remain unknown. In contrast to UDCA and TUDCA, which promote tubule-vesicular transport in hepatocytes [6], [25], [26], earlier studies have indicated that DHCA increases the canalicular bile flow via a microtubule-independent mechanism [15]. Accordingly, it was not surprising that colchicine did not modify the intrinsic and secretin-insensitive hydrocholeretic action of DHCA in our *in vivo* model. On the other hand, the concerted action of secretin and TUDCA to promote hydrocholeresis was sensitive to colchicine. Previous data indicating that secretin effects involve cAMP-stimulated vesicle mobilization [4], [27] suggest that colchicine targets mainly the action of secretin. Secretin signaling transduction is also known to result in increased intracellular cAMP levels and PKA and ERK1/2 activation [1], [28]. Thus, our finding that the PKA inhibitor H89 blocked the accelerated hydroionic expansion of cholangiocyte cystic structures in the presence of secretin+TUDCA was as expected.

PKA signaling may positively interact with other signal transduction cascades like Ca^2+^ signaling and activation of Ca^2+^-dependent PKCα, which appears to be closely associated with the effects of TUDCA in the hepatocytes [6] and cholangiocytes [29]. Thus, a cooperative mechanism involving PKA and Ca^2+^-dependent PKCα was recently described for the hepatocellular effects of TUDCA in a model of taurolitocholate-induced canalicular cholestasis [20], in which a seemingly permissive PKA activity appears to be associated with a crucial PKCα activity for the anticholestatic action of TUDCA. A similar scenario may occur in the cholangiocytes for TUDCA to promote the hydrocholeretic effect of secretin. Our *in vivo* and *in vitro* data showing that the PKCα inhibitor Gö6976 blocks the [TUDCA+secretin]-stimulated hydroionic secretion, support the role of PKCα for the cooperative action of TUDCA in these biliary cells. The effect of the intracellular-Ca^2+^ chelator BAPTA blocking the TUDCA promotion of secretin-stimulated expansion of cholangiocyte cystic structures further corroborate the role of Ca^2+^ signaling.

In addition to Ca^2+^/PKCα signaling, other signaling pathways like MAPK/MEK and PI3K have previously been associated with the effects of TUDCA in both hepatocytes [6] and cholangiocytes [29]. TUDCA was found to prevent the loss of bile ducts and restore a deficient hydrocholeretic response to secretin in rats with simultaneous bile duct ligation and vagotomy, and these effects were associated not only with increased intracelullar Ca^2+^ and stimulated PKCα, but also with activation of MAPK and PI3K cascades [29]. On the other hand, TUDCA was shown in an isolated perfused rat liver (IPRL) model to stimulate ATP release into bile [30]. Biliary ATP may then activate P2Y receptors in cholangiocytes inducing a Ca^2+^/PKCα/PI3K-dependent secretion of Cl^−^ [31], [32], which ultimately can be exchanged with HCO_3_^−^ leading to bicarbonate-rich hydrocholeresis. Our *in vivo* and *in vitro* findings indicate that inhibition of MEK (with U0126) and PI3K (with wortmannin) blocks the cooperative effect of TUDCA for secretin-stimulated hydrocholeresis.

Both in our model of normal rats with biliary drainage and maintained bile-acid pool [3], and in rats with ductal hyperplasia [2], secretin-stimulated hydrocholeresis has been shown to be dependent on the Cl^−^/HCO_3_^−^ exchange, a transport activity which in rat cholangiocytes is mediated by Ae2 [3], [16]. Here we confirmed that Ae2 also participates in the hydroionic secretion that expands our 3D-cultured cholangiocyte cystic structures (Fig. 5). In fact, our findings with *Ae2* silencing strongly suggest that the cyst expansion stimulated by TUDCA and/or secretin entails bicarbonate-rich hydroionic secretion into the cyst lumen.

As summarized in Fig. 6, our *in vivo* and *in vitro* data indicate that secretin-stimulated hydrocholeresis in normal rats infused with UDCA involves, at least partially, taurine-conjugation and subsequent TUDCA-secretin interaction in cholangiocytes results in activation of PKCα, PI3K, MEK and PKA pathways. Moreover, the bicarbonate extruder Ae2 is also involved in the hydrocholeretic effect. Our findings may enable a better understanding of the benefits of UDCA for the restoration of the response to secretin in patients with PBC. Together with the recent reports that unconjugated UDCA stimulates canalicular secretion of GSNO and biliary ATP release [14], and that norUDCA/ and/or TnorUDCA might represent a complement to UDCA therapies [23], our present mechanistic data may favor the design of new therapeutic approaches to improve the functionality of the biliary epithelium in patients with cholestatic disorders. Indeed, two pilot studies were performed during the nineties in some few patients with PBC which suggested that treatment with TUDCA might be of benefit *versus* unconjugated UDCA alone [33], [34]. And previous reports indicated that dietary taurine supplementation increases the proportion of taurine-conjugation in bile [35], [36]. Taurine supplementation could therefore be considered to possibly modulate the therapeutic properties of UDCA/TUDCA in PBC. It may be noticed that taurine supplementation is currently being proposed as potentially adjuvant for the treatment of different pathologies such as diabetes mellitus and its complications, although this is based on a series of evidence indicating that taurine can influence a variety of cellular functions in addition to conjugation of bile acids [37].

## Supporting Information

Figure S1 **TUDCA accelerates the secretin-stimulated expansion of 3D-cultured cholangiocyte cystic structures in a dose-dependent manner.** (A) Representative images of cystic structures in the presence of different doses of TUDCA (i.e. 0, 50, 100 and 200 µM) during 60 min, and with secretin for the last 30 min. (B) The presence of TUDCA favored in a dose-dependent manner the stimulatory effect of secretin on cyst expansion. Data are shown as mean ± SEM.
(TIF)

Figure S2 **Taurocholic acid (TCA) did not further promote the secretin-stimulated expansion of 3D-cultured cholangiocyte cystic structures.** (A) Representative images of cystic structures incubated in the presence of TCA during 60 min, and with secretin for the last 30 min. (B) The presence of TCA did not accelerate the stimulatory effect of secretin on cyst expansion. Data are shown as mean ± SEM.
(TIF)

Figure S3 **Effect of different inhibitors on the expansion of 3D-cultured cholangiocyte cystic structures.** (A) Representative images of cystic structures incubated for 60 min with TUDCA, and with secretin for the last 30 min, either in the presence or the absence of inhibitors. (B) None of the inhibitors blocked the spontaneous expansion of cystic structures; data are shown as mean ± SEM.
(TIF)

Figure S4 **Knockdown experiments with siRNA indicate that Ae2 is involved in the expansion of 3D-cultured cholangiocyte cystic structures.** (A) Representative images showing that siRNAs are internalized in cholangiocyte cystic structures: *left*, light microscopy image; *right*, fluorescence image of an internalized Cy3-labeled siRNA in red color, merged with the image on the left (artificially colored in green). (B) Representative images of cholangiocyte cystic structures previously incubated for 24 hours with either control siRNA (an siRNA against human Ae2 mRNA which does not target rat Ae2 mRNA), rAe2 siRNA (against rat Ae2 mRNA), or none siRNA. Cystic structures were in the presence of TUDCA for 60 minutes, being supplemented with secretin for the last 30 minutes.
(TIF)
