# Supplementary figures and images for: Ursodeoxycholic Acid Is Conjugated with Taurine to Promote Secretin-Stimulated Biliary Hydrocholeresis in the Normal Rat
Source: PLoS One. 2011 Dec 14;6(12):e28717. doi: 10.1371/journal.pone.0028717 (PMC3237485; doi:10.1371/journal.pone.0028717)

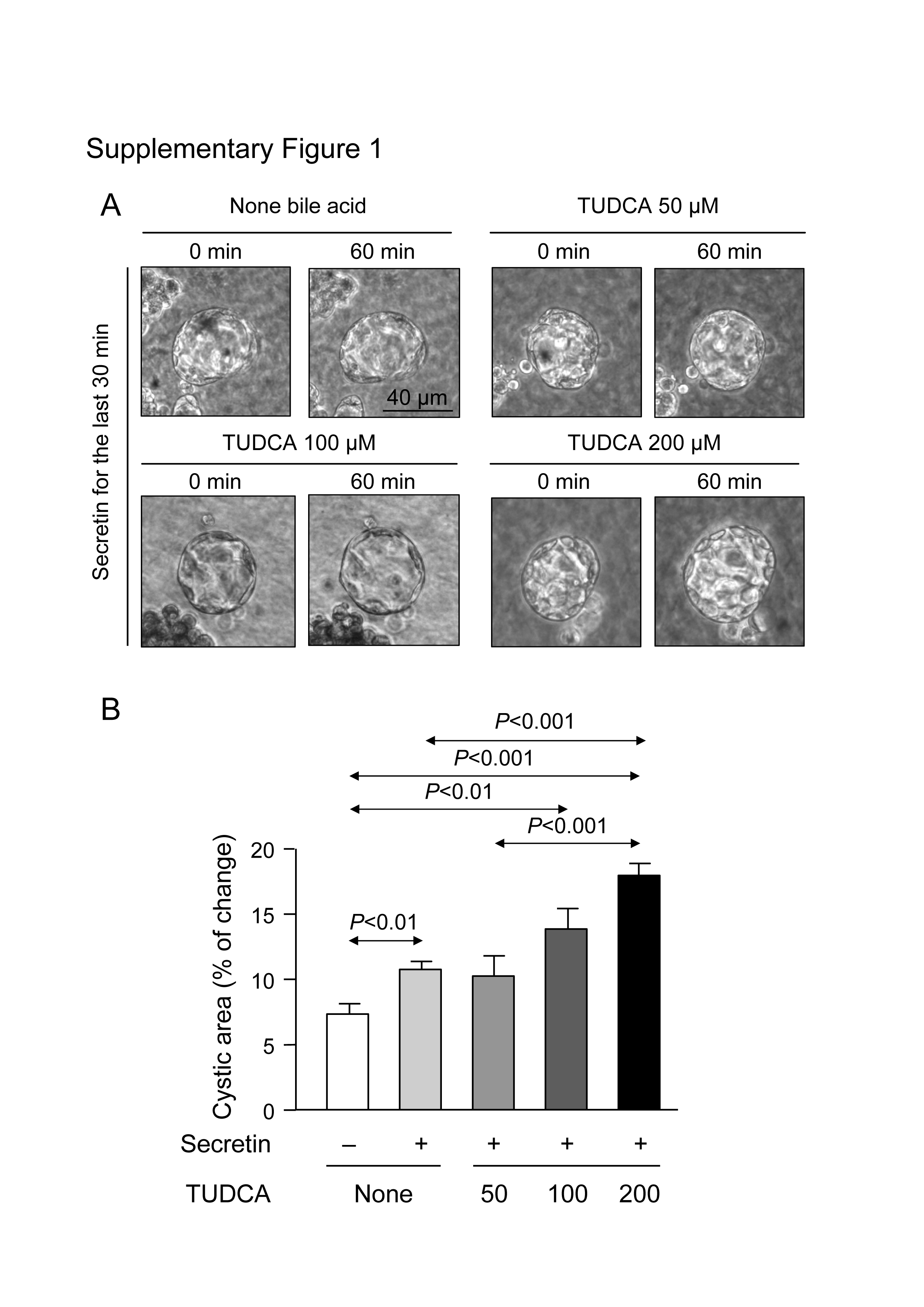

Supplement: Figure S1 — TUDCA accelerates the secretin-stimulated expansion of 3D-cultured cholangiocyte cystic structures in a dose-dependent manner. (A) Representative images of cystic structures in the presence of different doses of TUDCA (i.e. 0, 50, 100 and 200 µM) during 60 min, and with secretin for the last 30 min. (B) The presence of TUDCA favored in a dose-dependent manner the stimulatory effect of secretin on cyst expansion. Data are shown as mean ± SEM. (TIF) [file pone.0028717.s001.tif]

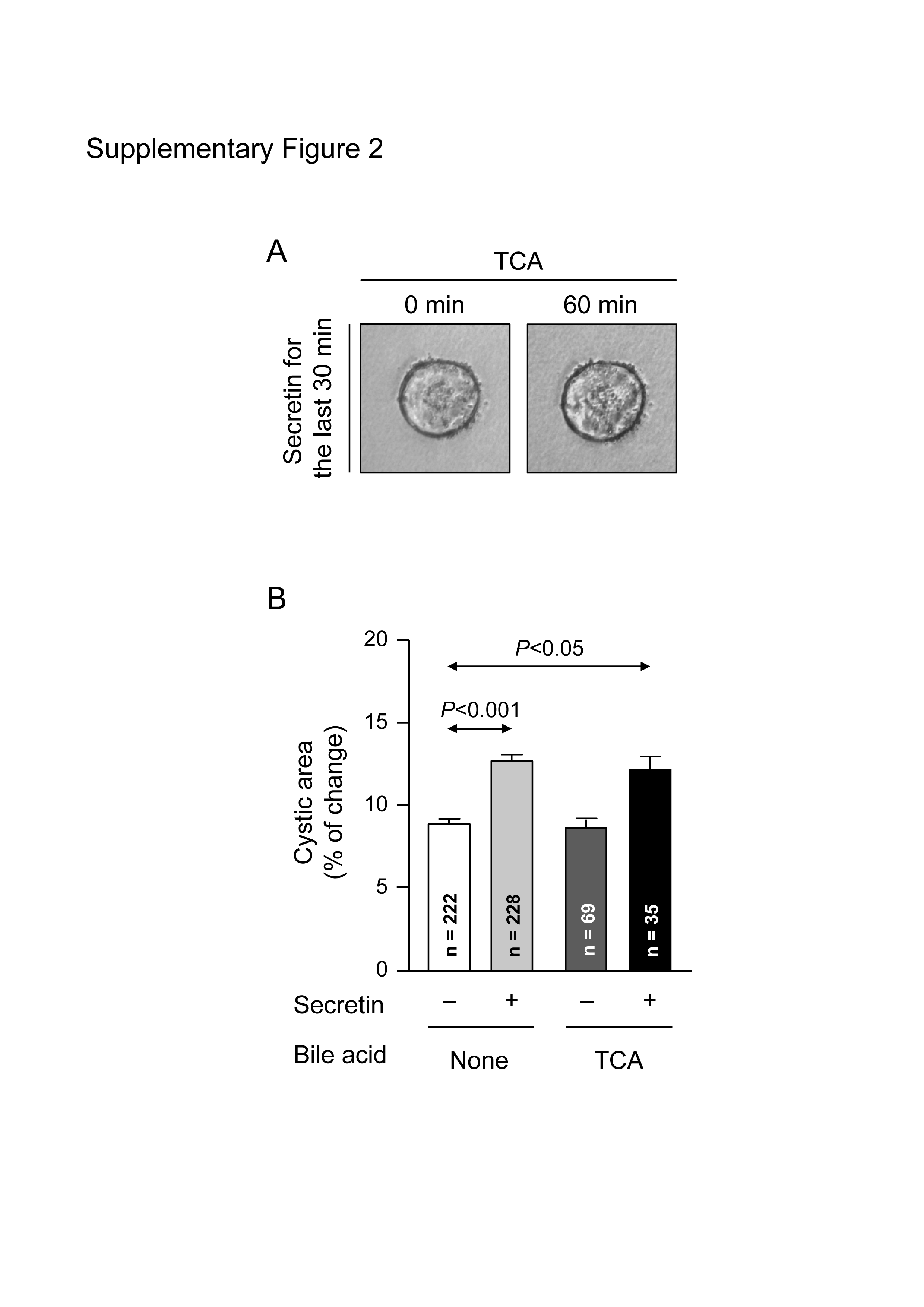

Supplement: Figure S2 — Taurocholic acid (TCA) did not further promote the secretin-stimulated expansion of 3D-cultured cholangiocyte cystic structures. (A) Representative images of cystic structures incubated in the presence of TCA during 60 min, and with secretin for the last 30 min. (B) The presence of TCA did not accelerate the stimulatory effect of secretin on cyst expansion. Data are shown as mean ± SEM. (TIF) [file pone.0028717.s002.tif]

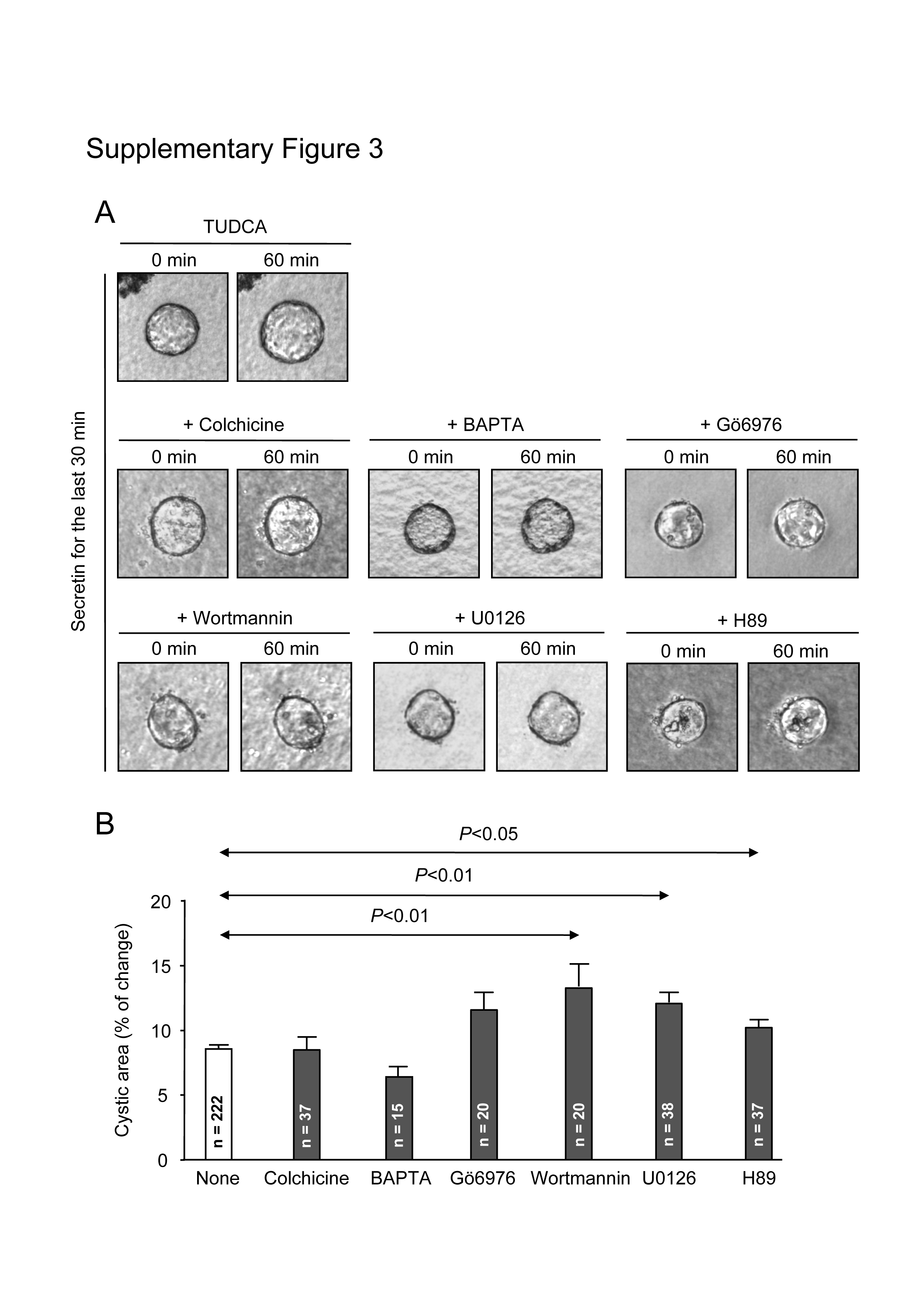

Supplement: Figure S3 — Effect of different inhibitors on the expansion of 3D-cultured cholangiocyte cystic structures. (A) Representative images of cystic structures incubated for 60 min with TUDCA, and with secretin for the last 30 min, either in the presence or the absence of inhibitors. (B) None of the inhibitors blocked the spontaneous expansion of cystic structures; data are shown as mean ± SEM. (TIF) [file pone.0028717.s003.tif]

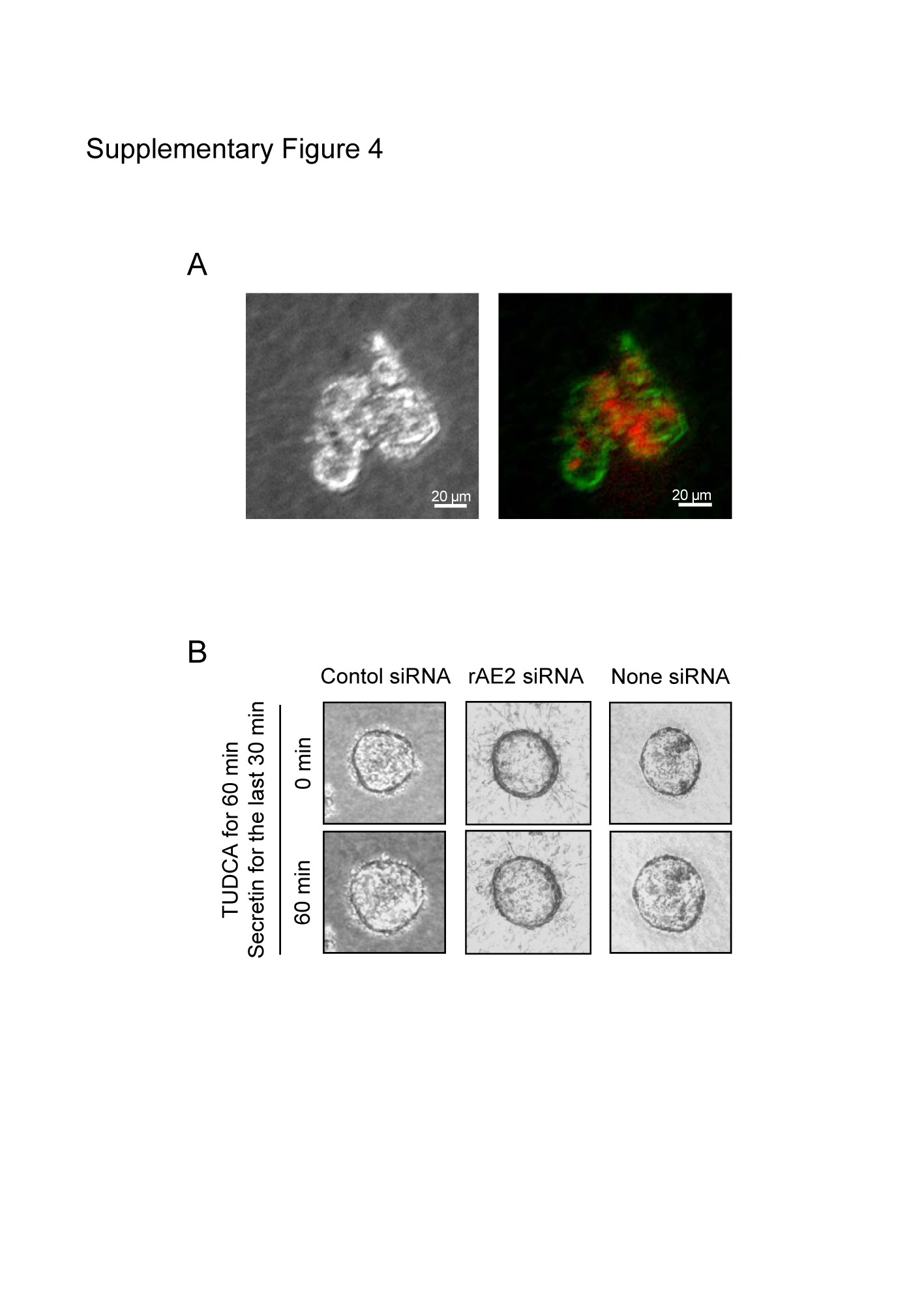

Supplement: Figure S4 — Knockdown experiments with siRNA indicate that Ae2 is involved in the expansion of 3D-cultured cholangiocyte cystic structures. (A) Representative images showing that siRNAs are internalized in cholangiocyte cystic structures: left, light microscopy image; right, fluorescence image of an internalized Cy3-labeled siRNA in red color, merged with the image on the left (artificially colored in green). (B) Representative images of cholangiocyte cystic structures previously incubated for 24 hours with either control siRNA (an siRNA against human Ae2 mRNA which does not target rat Ae2 mRNA), rAe2 siRNA (against rat Ae2 mRNA), or none siRNA. Cystic structures were in the presence of TUDCA for 60 minutes, being supplemented with secretin for the last 30 minutes. (TIF) [file pone.0028717.s004.tif]
